# Characterization of Rabbit Mesenchymal Stem/Stromal Cells after Cryopreservation

**DOI:** 10.3390/biology12101312

**Published:** 2023-10-07

**Authors:** Sai Koung Ngeun, Miki Shimizu, Masahiro Kaneda

**Affiliations:** 1 Laboratory of Veterinary Diagnostic Imaging, Faculty of Agriculture, Tokyo University of Agriculture and Technology, 3-5-8 Saiwai-cho, Fuchu, Tokyo 183-8509, Japan; s212892q@st.go.tuat.ac.jp; 2 Laboratory of Veterinary Anatomy, Faculty of Agriculture, Tokyo University of Agriculture and Technology, 3-5-8 Saiwai-cho, Fuchu, Tokyo 183-8509, Japan; kanedam@cc.tuat.ac.jp

**Keywords:** adipose tissues, bone marrow, differentiation potential, gene expression, surface marker

## Abstract

**Simple Summary:**

Mesenchymal stem cells (MSCs) are prime candidates for cell-based therapies and regenerative medicine due to their trilineage differentiation potential. MSCs can differentiate into osteocytes, adipocytes, and chondrocytes. A significant number of these cells must be available for clinical application. As a result, cryopreservation becomes essential to ensure the cells are readily available and can be stored long term. However, there is a gap in our knowledge concerning the characteristics, sustainability of multipotency, and biosafety of cryopreserved MSCs in rabbits. Additionally, the functional ability of MSCs varies based on their source, which could influence their cryopreservation response. Differences in morphology, differentiation potential, and resilience to cryopreservation might also exist among various animal models and humans. To learn more, we assessed MSCs’ characteristics and functional properties from rabbits before and after cryopreservation. These cells were sourced from adipose tissues and bone marrow. Our findings suggest that while bone marrow-derived MSCs retain their functional ability poorly, adipose-derived cells retain their functionality better.

**Abstract:**

Adipose tissues (ADPs) are an alternative source for mesenchymal stem/stromal cells (MSCs), given that conventional bone marrow (BM) collection is painful and yields limited cell numbers. As the need for easily accessible MSCs grows, cryopreservation’s role in regenerative medicine is becoming increasingly vital. However, limited research exists on the characteristics and functional properties of rabbit-derived MSCs from various anatomical sources before and after cryopreservation. We examined the effects of cryopreservation using Bambanker. We found that cryopreservation did not adversely affect the morphology, viability, and adipogenic or chondrogenic differentiation abilities of ADP MSCs or BM MSCs. However, there was a notable drop in the proliferation rate and osteogenic differentiation capability of BM MSCs post-cryopreservation. Additionally, after cryopreservation, the surface marker gene expression of *CD90* was not evident in ADP MSCs. As for markers, *ADIPOQ* can serve as an adipogenic marker for ADP MSCs. *ACAN* and *CNMD* can act as chondrogenic markers, but these two markers are not as effective post-cryopreservation on ADP MSCs, and osteogenic markers could not be validated. The study highlights that compared to BM MSCs, ADP MSCs retained a higher viability, proliferation rate, and differentiation potential after cryopreservation. As such, in clinical MSC use, we must consider changes in post-cryopreservation cell functions.

## 1. Introduction

Mesenchymal stem/stromal cells (MSCs) have shown promise in cellular therapy, tissue repair, and regenerative medicine research due to their therapeutic potential caused by their multipotent nature and self-renewal ability [1,2,3,4]. MSCs are also widely used in stem cell banks [5,6]. It is well known that MSCs can be isolated from bone marrow; however, they can also be isolated from adipose tissues (ADPs), dental pulp, umbilical cord blood, placenta, and many other adult tissues [7]. The process of sourcing MSCs should be non-invasive and comfortable, allow for the harvesting of large amounts of tissue, use tissue that has an abundance of MSCs, and maintain the MSCs’ viability and differentiation potential after long-term passaging and cryopreservation. The bone marrow harvesting procedure is painful, and the viability and differentiation potential of bone marrow-derived MSCs may decrease with donor age [8,9]. ADP-derived MSCs have attracted attention because fat harvesting is less invasive and uncomfortable than bone marrow harvesting, and MSCs in ADPs are numerous and easily isolated [10,11].

Cryopreservation is usually used for stem cell banking for future therapeutic purposes and to preserve animal genetic resources. The cells needed for cell-based therapy are available off the shelf [12], but if commercial products are not used, cells must be cryopreserved and expanded in vitro to ensure sufficient quantities of cells [13]. However, cryopreservation can cause reduced cell viability, molecular changes, and genetic and immunophenotypical alterations that can reduce the effectiveness of regenerative therapies [14,15]. On the other hand, there are reports that cryopreservation does not change the viability and functionality of cells [16,17].

Many studies have attempted to protect cells from damage during cryopreservation and maximize cell recovery by using cryoprotectants such as 10% DMSO or 1,2 propanediol [18,19]. The most common method is to use a cryoprotectant such as DMSO and preserve the cells in liquid nitrogen. However, Ock and Rho [20] reported that MSC viability was inversely proportional to DMSO concentration, highlighting the need for further investigation of the freezing medium. In addition, traditional cryopreservation methods have caused physical and molecular injuries to cells due to the complex freeze–thaw protocols [6]. Therefore, this study used Bambanker as the cell freezing medium, as an alternative to the conventional method, and cells were preserved at −80 °C [21].

Unlike rats and other rodents used as experimental animals, rabbits are suitable experimental animals as models for human bone regeneration medicine because they have Haversian canal structures in their bones and a similar bone regeneration process to humans [22,23,24,25,26]. Previous studies have reported species-related differences in the phenotype, surface markers, and gene expression of MSCs [4,27,28,29,30,31]. It has been demonstrated that the properties and characteristics of MSCs vary depending on the anatomical sources and donor [13,15,32,33]. Calle et al. [23] reported that rabbit MSCs from different tissues showed no differences in morphological features, differentiation potential, or intracellular and surface markers. However, the phenotype of rabbit MSCs varies between reports [5]. There are not as many studies on rabbit MSCs as for rats and humans [34,35,36]. Furthermore, the effects of cryopreservation on the functional stability and characteristics of the rabbit MSCs have not been adequately reported or compared between sources. 

Therefore, this study aimed to evaluate the characteristic and functional properties of cryopreserved rabbit ADP- and bone marrow-derived MSCs. We hypothesized that cryopreservation in Bambanker would reduce physical and molecular injury without altering the MSCs’ viability or functionality. We also hypothesized that ADP-derived MSCs are superior to bone marrow-derived MSCs because the collection process is non-invasive and comfortable, large amounts of tissue can be harvested, and the MSCs’ viability and differentiation potential are maintained after cryopreservation.

## 2. Materials and Methods

### 2.1. Experimental Animals and Experimental Groups

Four male New Zealand white rabbits aged 25.8 ± 2.5 months and with a body weight of 4.3 ± 0.3 kg were included in this experiment. The animal study protocol was approved by the Animal Care and Use Committee of the Tokyo University of Agriculture and Technology (approval numbers: R03-238 and R04-154). Rabbits were kept in cages at a room temperature of 18–23 °C with a 12 h light/dark cycle. They were fed 25 g/kg of laboratory animal dry food and water was provided ad libitum. At the end of the experiment, rabbits were euthanized by administering a high concentration of isoflurane until respiration ceased and death ensued. 

**Figure 1:**
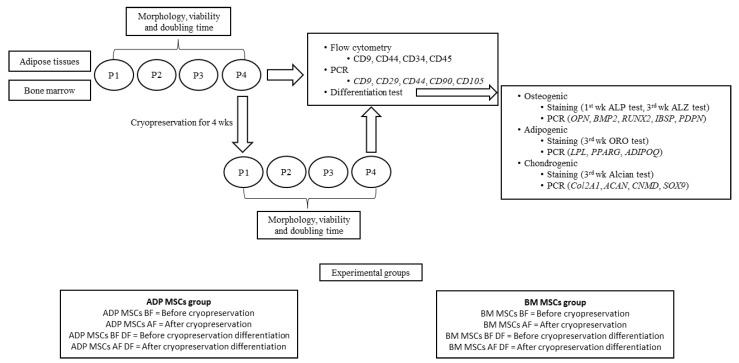
Experimental design. MSCs, mesenchymal stem/stromal cells; ADP, adipose tissue; BM, bone marrow; P1–4, passage 1–4.

Passage 4 cells harvested from ADPs were classified as the ADP MSCs group, and passage 4 cells harvested from bone marrow were classified as the BM MSCs group. The ADP and BM MSCs groups were each subdivided into four groups: before cryopreservation and before differentiation (the ADP MSCs BF and BM MSCs BF groups, respectively), before cryopreservation and after differentiation (the ADP MSCs BF DF and BM MSCs BF DF groups, respectively), after cryopreservation and before differentiation (the ADP MSCs AF and BM MSCs AF groups, respectively), and after cryopreservation and after differentiation (the ADP MSCs AF DF and BM MSCs AF DF groups, respectively). Figure 1 summarizes the experimental design.

### 2.2. Isolation and Culture of MSCs from ADP and Bone Marrow

Animals were anesthetized to perform ADP and bone marrow collection via an intramuscular injection of 1.5 mg/kg butorphanol, 1 mg/kg midazolam, and 0.25 mg/kg medetomidine as a general anesthesia [37]. Additionally, 50 mL/kg of lactated Ringer’s solution was administered subcutaneously as a fluid replacement.

ADPs (5 g) were collected from the subcutaneous fat of the scapular region under sterile conditions [38]. The collected ADPs were transferred to the laboratory in a tube containing PBS. After washing with PBS, samples were placed in a 60 mm diameter sterile culture dish (catalog no. TR4001, Nippon Genetics Co, Ltd., Tokyo, Japan) in a safety cabinet and minced using sterile scissors. Then, the minced ADPs were agitated in a shaking water bath for 1 h at 37 °C in Hank’s balanced salt solution (HBSS) (catalog no. 14025-092, Thermo Fisher Scientific Inc., New York, NY, USA) containing 0.1% collagenase type 1 (catalog no. SCR103, Sigma-Aldrich, St. Louis, MO, USA). DMEM containing 20% FBS was added to neutralize the collagenase enzyme activity. Solid aggregates were removed via filtration through a 100 µm filter. Then, cells were centrifuged at 800 g for 10 min, the supernatant fluid was removed, and 1 mL of RBC lysis buffer was used to resuspend the pellets and lyse the RBCs. After incubation with the RBC lysis buffer for 10 min, cells were washed with 10 mL of PBS. Following centrifugation at 600 g for 3 min, the supernatant fluid was discarded, and the cell pellets were resuspended in DMEM containing 20% FBS, 1% non-essential amino acids, and 1% Penicillin/Streptomycin as a basal culture medium. When the confluency reached 80%, cells were subcultured to the next passage.

Bone marrow (5 mL) was aspirated from the posterior iliac crest under sterile conditions using a bone marrow needle (18 gauge, 14–38 mm, Illinois bone marrow aspiration/intraosseous infusion needle, Nippon Becton Dickinson Company, Ltd., Tokyo, Japan) [39]. The bone marrow samples were passed through the RBC lysis protocol to purify them and remove the unwanted blood cells. Following vortexing for a few seconds in the RBC lysis buffer (catalog no. 60-00051-10, pluriSelect Life Science UG (haftungsbeschränkt) & Co. KG, Leipzig, Germany), the samples were stored at 4 °C for 10 min. After centrifugation at 300 g for 10 min, the supernatant fluid was discarded, and the cell pellets were washed with phosphate-buffered saline (PBS) (catalog no. 09-8912-100, Medicago AB, Uppsala, Sweden). Then, cell pellets were plated in Dulbecco’s Modified Eagle Medium (DMEM) (catalog no. 043-30085, FUJIFILM Wako Pure Chemical Corporation, Osaka, Japan) containing 20% fetal bovine serum (FBS) (catalog no. CCP-FBS-BR-500, COSMO BIO, Tokyo, Japan), 1% non-essential amino acids (catalog no. 139-15651, FUJIFILM Wako Pure Chemical Corporation, Osaka, Japan), and 1% Penicillin/Streptomycin (catalog no. 161-23181, FUJIFILM Wako Pure Chemical Corporation, Osaka, Japan) as a basal culture medium. When the confluency reached 80%, cells were subcultured to the next passage. 

Cells were cultured and passaged until passage 4 was reached for the before (the ADP MSCs BF and BM MCs BF) and after cryopreservation (the ADP MSCs AF and BM MSCs AF) groups.

### 2.3. Cryopreservation and Re-Culture of MSCs

One million passage 4 cells per cryopreserved vial (FastGene 2 mL cryotube) (catalog no. FG-CRY-In-20S, Nippon Genetics Co, Ltd., Tokyo, Japan) were cryopreserved in 1 mL of Bambanker (catalog no. CS-02-001, GC LYMPHOTEC Inc., Tokyo, Japan) at −80 °C. After 4 weeks of cryopreservation, cells were thawed at 37 °C in a water bath and immediately washed with PBS. After centrifugation, cell pellets were seeded in DMEM containing 20% FBS, 1% non-essential amino acids, and 1% Penicillin/Streptomycin and cultured again until passage 4 was reached. 

### 2.4. Cell Morphological and Viability Analysis

Cells morphology and viability were analyzed through inverted phase-contrast microscopy (CKX31, Olympus, Tokyo, Japan) at ×40 magnification [40] for the four groups of MSCs in passage 4: ADP MSCs BF, BM MSCs BF, ADP MSCs AF, and BM MSCs AF. To calculate cell viability and doubling time, the total cells were counted using the Hirschmann counting chamber THOMA (Code no. 8100105, Hirschmann Laborgeräte GmbH & Co. KG, Eberstadt, Germany) at each passage, and the percentage of live cells was calculated using the trypan blue exclusion method (catalog no. 204-21102, FUJIFILM Wako Pure Chemical Corporation, Osaka, Japan). Both viability and doubling time were used to define proliferative performance. The doubling time was calculated using the duration between passages (day) and the number of viable cells with the following formula. Medians of the cell doubling times and viability of four passages were compared between groups.

$$Doublingtime=\frac{Duration\times \log\left(2\right)}{log(finalconcentration)-\log\left(initialconcentration\right)}$$

### 2.5. Evaluation of Immunophenotypic Characterization on MSCs

In passage 4 for each of the four groups (ADP MSCs BF, ADP MSCs AF, BM MSCs BF, and BM MSCs AF), the MSCs were subjected to flow cytometry and RT-PCR. 

Standard MSC surface markers and hematopoietic cell markers were detected by flow cytometry. CD9 (catalog no. NBP1-28364, Novus Biologicals, Centennial, CO, USA) and CD44 (catalog no. bs-0521R-FITC, Bioss, Woburn, MA, USA) were used as MSC surface marker antibodies, and CD34 (catalog no. bs-0646R-FITC, Bioss, Woburn, MA, USA) and CD45 (catalog no. MHCD450, Thermo Fisher Scientific Inc., Life Technologies Corporation, Frederick, MD, USA) were used as hematopoietic cell marker antibodies [40,41,42,43,44]. For the negative control, rabbit IgG isotype control (catalog no. bs-0295p-fitc, Bioss, Woburn, MA, USA) and mouse IgG isotype control (catalog no. bs-0296P-FITC, Bioss, Woburn, MA, USA) were used. Cells were washed three times with HBSS, and the cell concentrations were adjusted to 1 × 10^6^ cells/mL. The cell suspensions were incubated with the respective FITC-labeled antibodies at the concentration stated in the manufacturer’s instructions for 20 min in darkness at 4 °C. The unbound antibodies were washed off using HBSS. Cell surface antigens were detected, and their expression percentages were examined using a flow cytometer (Beckman Coulter, Brea, CA, USA) and analyzed with CytExpert Software v1.2 (Beckman Coulter, Brea, CA, USA).

The gene expression of surface markers was confirmed at the mRNA level with RT-PCR by using the MSCs’ surface marker-specific primers of *CD9*, *CD29*, *CD44*, *CD90*, and *CD105*. Total RNA was extracted using the FastGene RNA Basic Kit (catalog no. FG-80250, Nippon Genetics Co., Ltd., Tokyo, Japan) according to the manufacturer’s instructions. To remove the DNA contamination, the extracted RNA was treated with the TURBO DNA-free™ Kit (catalog no. AM1907, Thermo Fisher Scientific Inc., Vilnius, Lithuania). RNA quantity and purity were evaluated with a NanoDrop^TM^ Lite spectrophotometer (catalog no. ND-LITE-PR, Thermo Fisher Scientific Inc., Wilmington, DE, USA). The first-strand cDNA was synthesized using a reverse transcription kit, the ReverTra Ace^®^ qPCR RT Master Mix (code no. FSQ-201, Toyobo Inc., Osaka, Japan), according to the manufacturer’s instructions. Primers used for the PCR reactions are shown in Table 1. After the PCR was set up with 1 μL of the respective primer, 2 μL of each cDNA, 7 μL of distilled water, and 10 μL of EmeraldAmp^®^ PCR Master Mix (catalog no. RR300A, Takara Bio Inc., Shiga, Japan), amplification was performed with initial denaturation at 95 °C for 5 min, followed by 30 cycles of denaturation at 95 °C for 30 s, annealing at 50–65 °C for 30 s, and extension at 72 °C for 30 s. After amplification, 6 μL of the reaction mixture was subjected to electrophoresis and analyzed on 2% agarose gel for 40 min. The bands were visualized by a UV light chamber (AE-6932GXESCP-1 and ATTO WiseUV^®^, ATTO Corporation, Tokyo, Japan). The RNA and cDNA were stored at −80 °C and −20 °C, respectively, for later use. Glyceraldehyde-3-phosphate dehydrogenase was used as a reference gene for internal reaction control. The number of cells expressing MSC surface marker genes was measured.

### 2.6. Evaluation of the Differentiation Ability

Differentiation potential is one way to evaluate the multipotentiality of MSCs. Plastic-adherent MSCs in passage 4 in the four groups (ADP MSCs BF, ADP MSCs AF, BM MSCs BF, and BM MSCs AF) were induced by the specific induction medium and evaluated for osteogenic, adipogenic, and chondrogenic differentiation capacity. Differentiated cells were confirmed by tissue-specific staining and lineage-specific gene expression analysis with RT-PCR. 

Tissue-specific staining rates were analyzed with ImageJ (https://imagej.nih.gov/ij/download.html (8 January 2022)) to determine the percentage of stained cells and the optical density within the observation area. The basal medium was used as a negative control for the before-differentiation groups. The after-differentiation staining percentage was calculated by subtracting the before-differentiation staining rate from the after-differentiation staining rate.

#### 2.6.1. Osteogenic Differentiation

In a 24-well plate (catalog no. TR5002, Nippon Genetics Co, Ltd., Tokyo, Japan), cells were counted and seeded at a density of 2 × 10^4^ per well. At 80% confluence, the osteogenesis differentiation medium (DMEM supplemented with 20% FBS and 100 nM dexamethasone, catalog no. D4902, Sigma-Aldrich, St. Louis, MO, USA), 0.2 mM ascorbic acid (catalog no. 016-04805, FUJIFILM Wako Pure Chemical Corporation, Osaka, Japan), and 10 mM b-glycerol phosphate (catalog no. 17130-22, Nacalai Tesque, Inc., Kyoto, Japan) were added. The medium was renewed every three days for 21 days. Osteogenic differentiation ability was identified via alkaline phosphatase (ALP) activity and Alizarin Red S (ALZ) staining [42]. The stained percent (%) within 6 mm^2^ of the observation area was analyzed once for each sample (*n* = 4) with ImageJ. 

##### Alkaline Phosphatase (ALP) Activity

ALP activity was analyzed after 7 days of culture. Cell layers were washed twice with PBS, fixed with 4% paraformaldehyde for 10 min at 4 °C, rinsed with PBS, and stained with 5-bromo-4-chloro-3-indolyl phosphate (catalog no. B5655-5TAB, Sigma-Aldrich, St. Louis, MO, USA) for 2 h in darkness at room temperature. The chromogenic reactions were stopped by washing the samples twice with dH_2_O. Following drying, the samples were observed under a light microscope (CKX31, Olympus, Tokyo, Japan) at ×40 magnification. Alkaline phosphatase activity due to osteoblast differentiation was stained a dark purple color. Cells exhibiting ALP activity were identified by culture wells stained a purple-blue color. The stained percent (%) within 6 mm^2^ of the observation area was analyzed once for each sample (*n* = 4) with ImageJ.

##### Alizarin Red (ALZ) Staining

ALZ staining was performed after the differentiation of cells for 21 days in the induction media. Cells were washed twice with PBS, fixed with ice-cold 70% ethanol for 1 h at 4 °C, and then rinsed twice with dH_2_O. ALZ solution (catalog no. 40-1009-5, Sigma-Aldrich, St. Louis, MO, USA) was added to cover the cells, which was followed by incubation at room temperature for 30 min. The wells were washed four times with dH_2_O, and images were taken using an inverted microscope (CKX31, Olympus, Tokyo, Japan) at ×40 magnification. Red staining of the mineralized matrix confirmed the osteogenic differentiation. The stained percent (%) within 6 mm^2^ of the observation area was analyzed once for each sample (*n* = 4) with ImageJ.

##### Gene Expression in Osteogenic Cells

Expression of the osteogenic-related genes osteopontin (*OPN*), integrin-binding sialoprotein (*IBSP*), bone morphogenetic protein-2 (*BMP2*), runt-related transcription factor 2 (*RUNX2*), and podoplanin (*PDPN*) was analyzed through RT PCR and described as the number of rabbits in which expression was detected.

#### 2.6.2. Adipogenic Differentiation

In a 24-well plate, cells were counted and seeded at a density of 2 × 10^4^ per well. At 80% confluence, an adipogenic-induction medium containing DMEM supplemented with 20% FBS, 1 µM dexamethasone, 500 µM isobutylmethylxanthine (catalog no. AG-CR1-3512-G001, Adipogen Life Science Inc., San Diego, CA, USA), 100 µM indomethacin (catalog no. 405268, Sigma-Aldrich), and 5 µg/mL insulin (catalog no. 16634, Sigma-Aldrich, St. Louis, MO, USA) were added. The medium was renewed every 3 days for 21 days.

##### Oil Red O Staining

After 21 days, the cells were washed with PBS, fixed with 4% paraformaldehyde for 15 min, and stained with 0.5% Oil Red O (catalog no. O-0625, Sigma-Aldrich, St. Louis, MO, USA) in isopropanol–distilled water (3:2) for 10 min to detect intracellular lipid accumulation. Adipogenic differentiation was detected using a light microscope (CKX31, Olympus) at ×100 magnification. The accumulation of red-stained lipids in the cells by Oil Red O staining confirmed the adipocytes. The stained percent (%) within 1 mm^2^ of the observation area was analyzed once for each sample *(n* = 4) with ImageJ.

##### Gene Expression in Adipogenic Cells

Expression of the adipogenic-related genes lipoprotein lipase (*LPL*), peroxisome proliferator-activated receptor gamma (*PPARG*), and adiponectin (*ADIPOQ*) was analyzed through RT PCR and described as the number of rabbits in which expression was detected.

#### 2.6.3. Chondrogenic Differentiation

Cells (2 × 10^4^) were resuspended in an inductor medium (DMEM supplemented with 20% FBS, 100 nM dexamethasone, 50 µg/mL ascorbic acid, and 10 ng/mL transforming growth factor (TGF) β3 (catalog no. HZ1090, Proteintech Group, Inc., Rosemont, IL, USA) and 1% premix ITS (catalog no. 354352, Corning Inc., New York, NY, USA), which contains insulin, human transferrin, sodium selenite, and 40 µg/mL L-proline (catalog no. P0481, Tokyo Chemical Industry Co. Ltd., Tokyo, Japan), seeded in a 24-well plate, and maintained at 37 °C in a humidified atmosphere with 5% CO_2_. The culture medium was changed twice a week.

##### Alcian Blue Staining

After 21 days, cells were stained with Alcian Blue stain to evaluate chondrogenesis under a light microscope. For this purpose, cells were washed twice with PBS and fixed with 4% paraformaldehyde for 30 min at room temperature. Then, cells were washed with dH_2_O and incubated overnight with 1% Alcian Blue (catalog no. 66011-100ML-F, Sigma-Aldrich, St. Louis, MO, USA) at room temperature and protected from direct light. The following day, cells were removed from the staining solution and washed 2–3 times with 0.1 N hydrochloric acid (HCL) (product code 083-01115, FUJIFILM Wako Pure Chemical Corporation, Osaka, Japan). Subsequently, the HCL solution was removed, dH_2_O was added, and cells were observed under a light microscope (CKX31, Olympus, Tokyo, Japan) at ×40 magnification. Alcian Blue staining was used to assess the chondrogenic differentiation by staining for highly sulfated proteoglycans in the cartilage matrix. The stained color’s optical density within 6 mm^2^ of the observation area was analyzed once for each sample (*n* = 4) with ImageJ.

##### Gene Expression in Chondrogenic Cells

Expression of the chondrogenic-related genes collagen type II alpha 1 chain (*COL2A1*), aggrecan (*ACAN*), chondromodulin (*CNMD*), and SRY-box 9 (*SOX9*) was analyzed via RT PCR and described as the number of rabbits in which expression was detected.

### 2.7. Statistical Analysis

All data except the PCR results were subjected to statistical analysis using GraphPad Prism software version 9 (GraphPad Software, Inc., La Jolla, CA, USA). Data not following a normal distribution are presented as medians, and between-group comparisons were performed using the Kruskal–Wallis test followed by Dunn’s multiple comparison test. The level of significance was set at *p* < 0.05. 

## 3. Results

### 3.1. Isolation and Morphology of Rabbit MSCs

**Figure 2:**
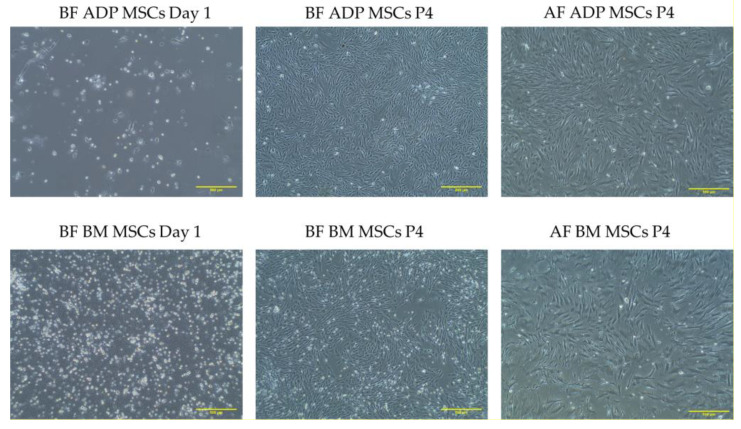
Cell morphology of plastic-adherent rabbit ADP and BM MSCs in day 1 culture and 4th passage before and after cryopreservation. The scale bar represents 500 µm. MSCs, mesenchymal stem/stromal cells; ADP, adipose tissue; BM, bone marrow; BF, before cryopreservation; AF, after cryopreservation; P4, passage 4.

After 24 h of culture (day 1), plastic-adherent MSCs were predominantly circular-shaped, which was followed by the appearance of spindle-shaped fibroblast-like cells, the typical morphology of MSCs. Their morphology had no distinct variation after cryopreservation (Figure 2). 

### 3.2. Viability and Doubling Time

**Figure 3:**
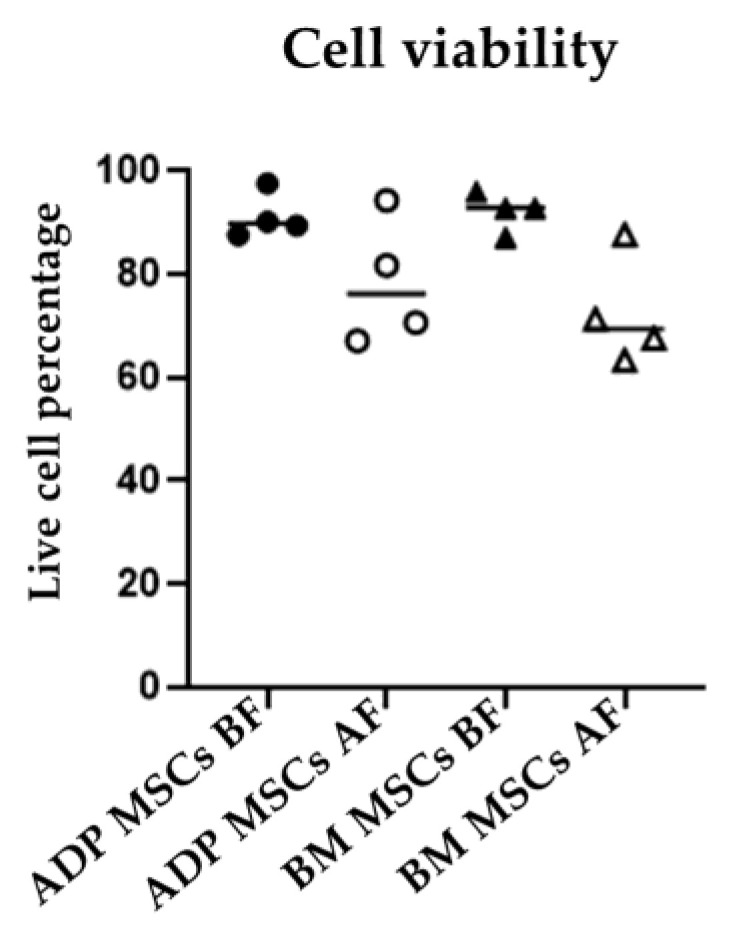
Cell viability of MSCs before and after cryopreservation. The live cell percentage of ADP MSCs and BM MSCs did not change significantly after cryopreservation. Data were analyzed with the Kruskal–Wallis test and presented as the median (*n* = 4). MSCs, mesenchymal stem/stromal cells; ADP, adipose tissue; BM, bone marrow; BF, before cryopreservation; AF, after cryopreservation; black circle, ADP MSCs BF; white circle, ADP MSCs AF, black triangle, BM MSCs BF; white triangle, BM MSCs AF, horizontal line, median.

**Figure 4:**
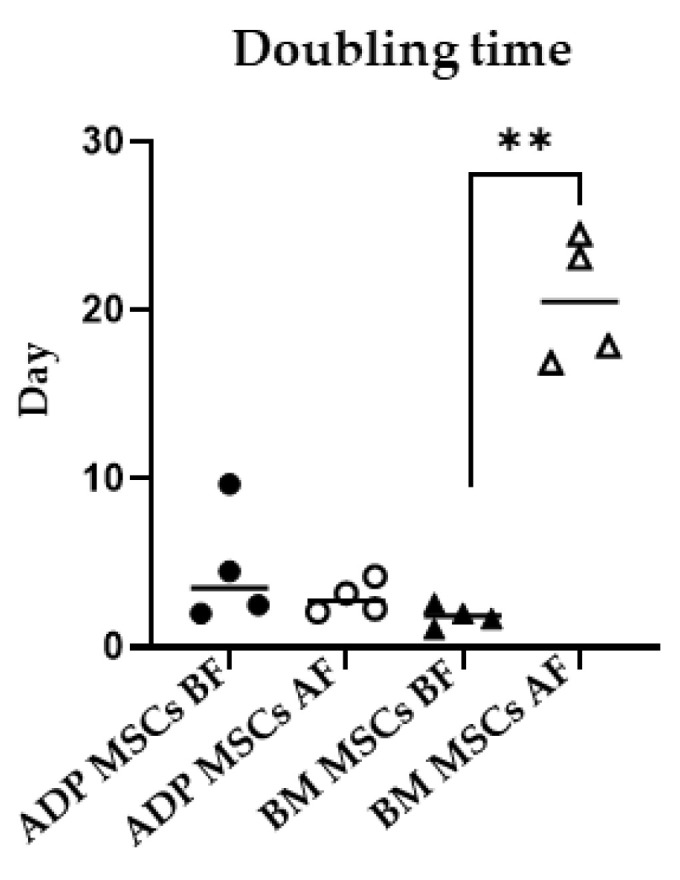
Cell doubling time of MSCs before and after cryopreservation. Cell doubling times in the BM MSCs AF group increased significantly more than the BM MSCs BF group (** *p* < 0.01), while doubling times of ADP MSCs remained unchanged. Data were analyzed with the Kruskal–Wallis test, followed by Dunn’s multiple comparison test, and presented as the median (*n* = 4). MSCs, mesenchymal stem/stromal cells; ADP, adipose tissue; BM, bone marrow; BF, before cryopreservation; AF, after cryopreservation; black circle, ADP MSCs BF; white circle, ADP MSCs AF, black triangle, BM MSCs BF; white triangle, BM MSCs AF, horizontal line, median.

The median (range) viability (%) in the ADP MSCs BF and BM MSCs BF was 89.8 (87.5–97.5) and 92.8 (87.1–96.0), respectively. After cryopreservation, the median (range of) viability (%) in the ADP MSCs AF and BM MSCs AF was 76.2 (67.1–94.3) and 69.4 (63.3–87.6), respectively. There was no difference in cell viability among the four groups (Figure 3). The median (range of) doubling time (days) in the ADP MSCs BF and BM MSCs BF was 3.5 (2.0–9.7) and 1.9 (1.1–2.6), respectively. After cryopreservation, the median (range of) doubling time (days) in the ADP MSCs AF and BM MSCs AF was 2.8 (2.1–4.2) and 20.5 (16.9–24.5), respectively. Doubling time was increased in the BM MSCs AF group compared to the BM MSCs BF group (*p* = 0.0021) (Figure 4).

### 3.3. Surface Marker Expression of MSCs Determined with Flow Cytometry and RT-PCR 

**Figure 5:**
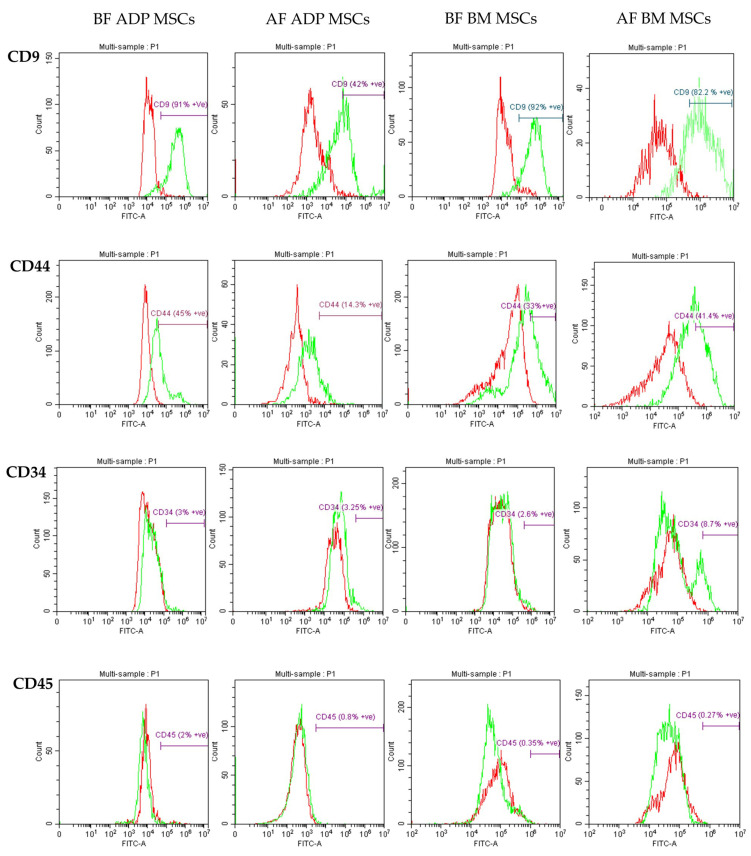
Representative histograms of MSC surface marker expression of CD9 and CD44, and hematopoietic marker expression of CD34 and CD45. The red histogram represents isotype control, and the green histogram represents respective antibodies. MSCs, mesenchymal stem/stromal cells; ADP, adipose tissue; BM, bone marrow; BF, before cryopreservation; AF, after cryopreservation.

The percentage of cell surface marker expression detected via flow cytometry is presented as the median (range) and shown in Table 2. The representative histograms of surface marker expression are shown in Figure 5. In all groups, the expression of the MSC-specific markers CD9 and CD44 was high; however, the CD9 expression percentage of the ADP MSCs AF group was lower compared to that of the ADP MSCs BF group (*p* = 0.0401). The expression of the hematopoietic cell markers CD34 and CD45 was low. 

**Figure 6:**
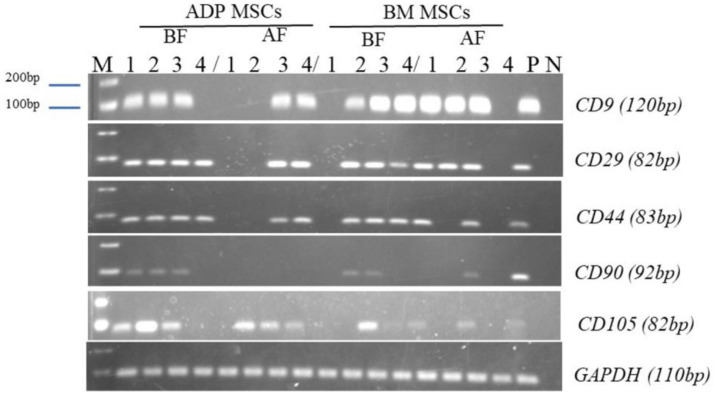
RT-PCR analysis of surface marker genes’ expression in the 4th passage of MSCs before and after cryopreservation. The numbers 1–4 are the individual numbers of the four rabbits; M, marker (DNA ladder, between 100–200 bp); P, positive control; N, negative control; MSCs, mesenchymal stem/stromal cells; ADP, adipose tissue; BM, bone marrow; BF, before cryopreservation; AF, after cryopreservation.

The data of the cell surface marker gene expression detected via RT-PCR are shown in Figure 6, and the number of rabbits in which expression was detected is shown in Table 3. The MSC surface marker genes were highly expressed before cryopreservation (the ADP MSCs BF and BM MSCs BF groups). However, gene expression tended to decrease after cryopreservation.

### 3.4. Differentiation Potential of Rabbit MSCs 

#### 3.4.1. Osteogenic Differentiation 

**Figure 7:**
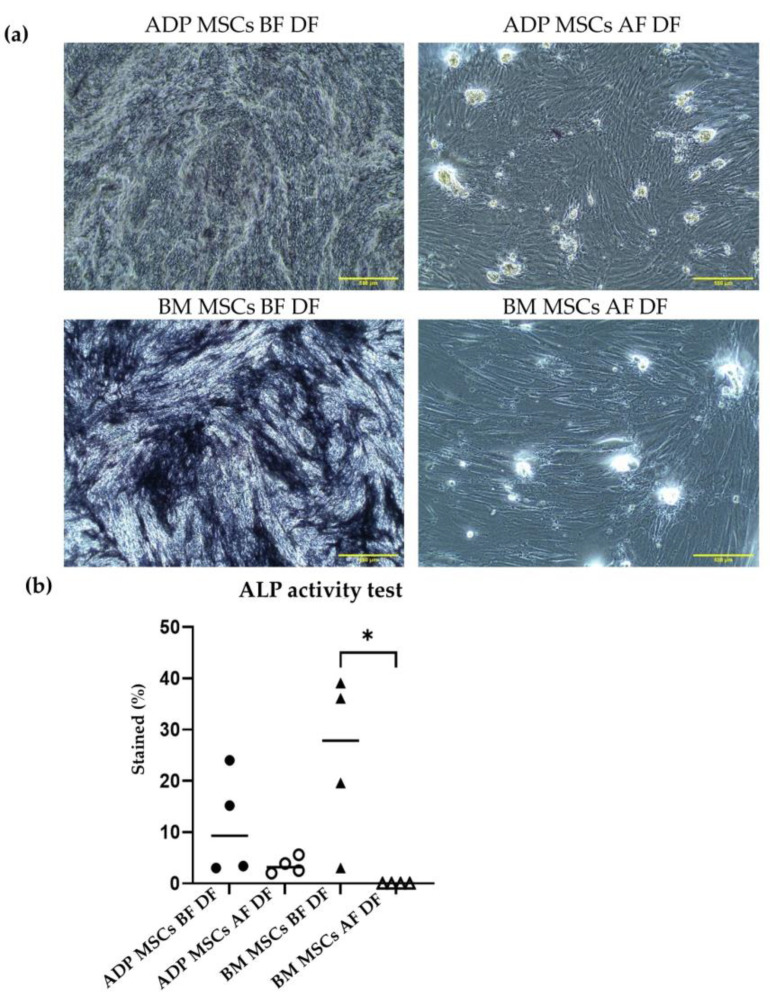
(**a**) Representative histological image of alkaline phosphatase staining of the induced osteogenic differentiated cells derived from rabbit ADP MSCs and BM MSCs before and after cryopreservation. The scale bar represents 500 µm. (**b**) The alkaline phosphatase-stained percent (%) within 6 mm^2^ of the observation area was analyzed with ImageJ. The stained % of the BM MSCs AF DF group was significantly lower compared to that of the BM MSCs BF DF group (* *p* < 0.05). Data were analyzed with the Kruskal–Wallis test, followed by Dunn’s multiple comparison test, and presented as the median (*n* = 4). MSCs, mesenchymal stem/stromal cells; ADP, adipose tissue; BM, bone marrow; BF, before cryopreservation; AF, after cryopreservation; DF, differentiated cells; black circle, ADP MSCs BF DF; white circle, ADP MSCs AF DF, black triangle, BM MSCs BF DF; white triangle, BM MSCs AF DF, horizontal line, median.

The median (range) stained percentage of ALP activity in the ADP MSCs BF DF and BM MSCs BF DF groups was 9.3 (3.0–24.0) and 27.9 (3.0–39.1), respectively. After cryopreservation, the median (range) stained percentage of ALP activity in the ADP MSCs AF DF and BM MSCs AF DF groups was 3.2 (2.0–5.6) and 0 (0–0), respectively. The stained percentage of ALP activity in the BM MSCs AF DF group was lower compared to that of the BM MSCs BF DF group (*p* = 0.0113) (Figure 7).

**Figure 8:**
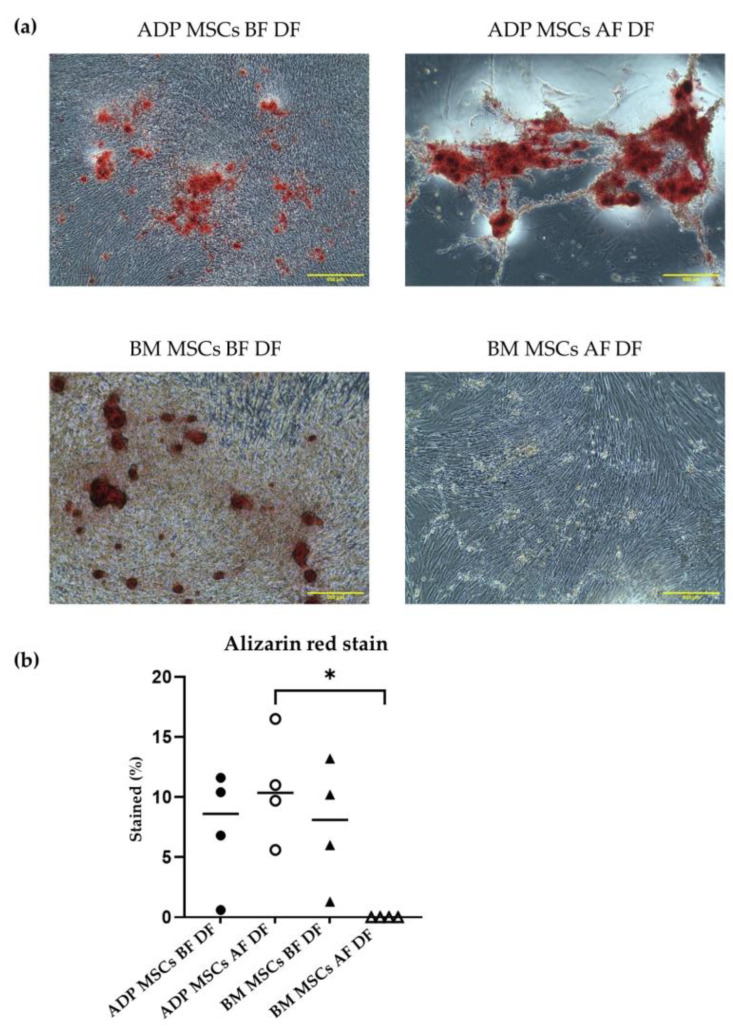
(**a**) Representative histological image of Alizarin Red staining of the induced osteogenic differentiated cells derived from rabbit ADP MSCs and BM MSCs before and after cryopreservation. The scale bar represents 500 µm, magnification × 40. (**b**) The Alizarin Red stained percent (%) of the mineral matrix deposition within 6 mm^2^ of the observation area was analyzed with ImageJ. The stained % of BM MSCs AF DF was significantly lower compared to that of ADP MSCs AF DF (* *p* < 0.05). Data were analyzed with the Kruskal–Wallis test, followed by Dunn’s multiple comparison test, and presented as the median (*n* = 4). MSCs, mesenchymal stem/stromal cells; ADP, adipose tissue; BM, bone marrow; BF, before cryopreservation; AF, after cryopreservation; DF, differentiated cells; black circle, ADP MSCs BF DF; white circle, ADP MSCs AF DF, black triangle, BM MSCs BF DF; white triangle, BM MSCs AF DF, horizontal line, median.

The median (range) stained percentage of ALZ in the ADP MSCs BF DF and BM MSCs BF DF groups was 8.6 (0.6–11.6) and 8.1 (1.3–13.2), respectively. After cryopreservation, the median (range) stained percentage of AlZ in the ADP MSCs AF DF and MB MSCs AF DF groups was 10.4 (5.6–16.5) and 0 (0–0), respectively. The stained percentage of ALZ stain in the BM MSCs AF DF group was lower compared to that of the ADP MSCs AF DF group (*p* = 0.0425) (Figure 8).

**Figure 9:**
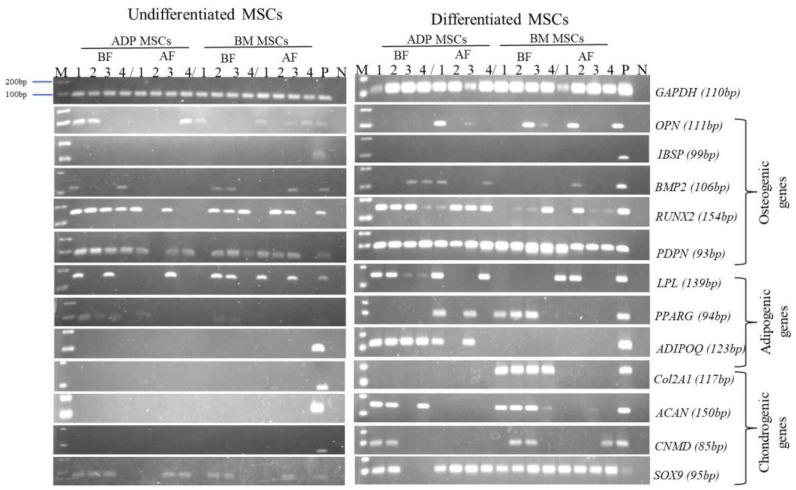
RT-PCR analysis of GAPDH as an internal control, and of osteogenic gene, adipogenic gene, and chondrogenic gene expression in the 4th passage of MSCs before and after cryopreservation. The numbers 1–4 are the individual numbers of the four rabbits; M, marker (DNA ladder, between 100–200 bp); P, positive control; N, negative control; MSCs, mesenchymal stem/stromal cells; ADP, adipose tissue; BM, bone marrow; BF, before cryopreservation; AF, after cryopreservation.

In the gene expression analysis carried out with RT-PCR after 21 days of differentiation (Table 4 and Figure 9), *IBSP* was not expressed in all groups. *OPN*, *BMP2*, *RUNX2*, and *PDPN* were expressed in undifferentiated MSCs.

#### 3.4.2. Adipogenic Differentiation Confirmed by Oil Red O Staining

**Figure 10:**
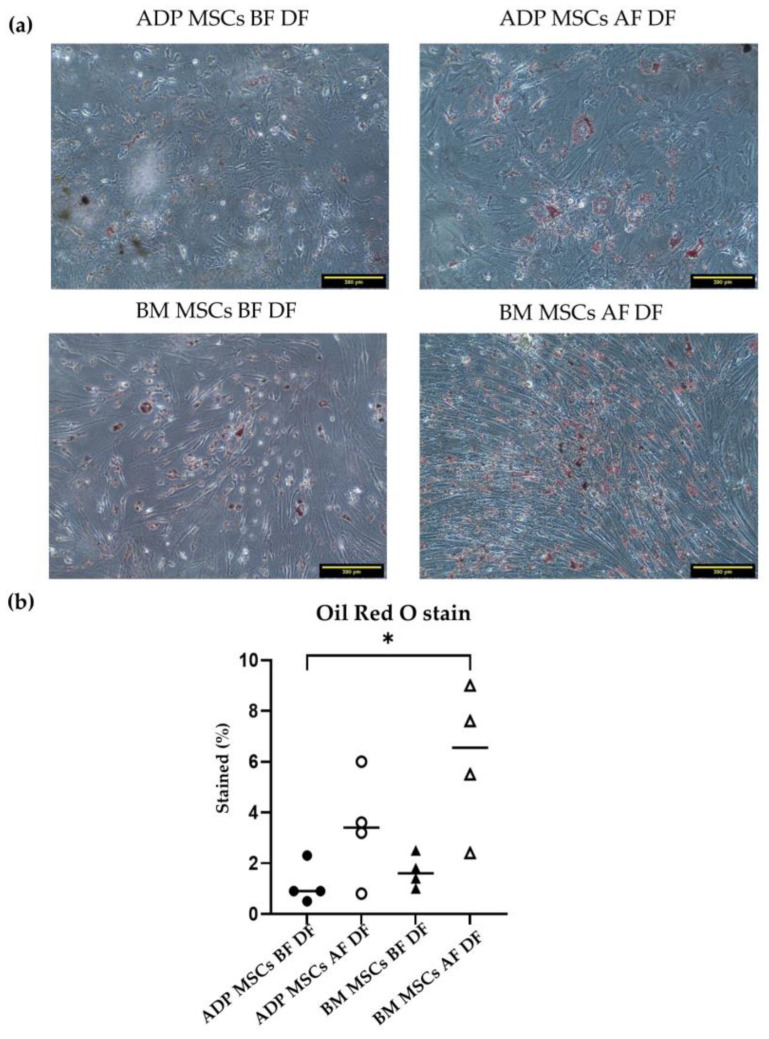
(**a**) Representative histological image of Oil Red O staining of the induced adipogenic differentiated cells derived from rabbit ADP MSCs and BM MSCs before and after cryopreservation. The scale bar represents 200 µm. (**b**) The Oil Red O-stained percent (%) of the fat vacuoles within 1 mm^2^ of the observation area was analyzed with ImageJ. The stained % of the BM MSCs AF DF group was significantly higher compared to that of the ADP BF DF MSCs group (* *p* < 0.05). Data were analyzed with the Kruskal–Wallis test, followed by Dunn’s multiple comparison test, and presented as the median (*n* = 4). MSCs, mesenchymal stem/stromal cells; ADP, adipose tissue; BM, bone marrow; BF, before cryopreservation; AF, after cryopreservation; DF, differentiated cells; black circle, ADP MSCs BF DF; white circle, ADP MSCs AF DF, black triangle, BM MSCs BF DF; white triangle, BM MSCs AF DF, horizontal line, median.

The median (range) stained percentage of Oil Red O in the ADP MSCs BF DF and BM MSCs BF DF groups was 0.9 (0.5–2.3) and 1.6 (1.0–2.5), respectively. After cryopreservation, the median (range) stained percentage of Oil red O in the ADP MSCs AF and MB MSCs AF groups was 3.4 (0.8–6.0) and 6.6 (2.4–9.0), respectively. The stained percentage of Oil Red O stain in the BM MSCs AF DF group was higher compared to that of the ADP MSCs BF DF group (*p* = 0.0358) (Figure 10). 

In the gene expression analysis carried out via RT-PCR after 21 days of differentiation (Table 4 and Figure 9), *ADIPOQ* was expressed in ADP MSCs before and after cryopreservation (the ADP MSCs BF DF and ADP MSCs AF DF groups) but not in the BM MSCs (the BM MSCs BF DF and BM MSCs AF DF groups). *LPL* and *PPARG* genes were expressed in undifferentiated MSCs.

#### 3.4.3. Chondrogenic Differentiation Confirmed by Alcian Blue Stain

**Figure 11:**
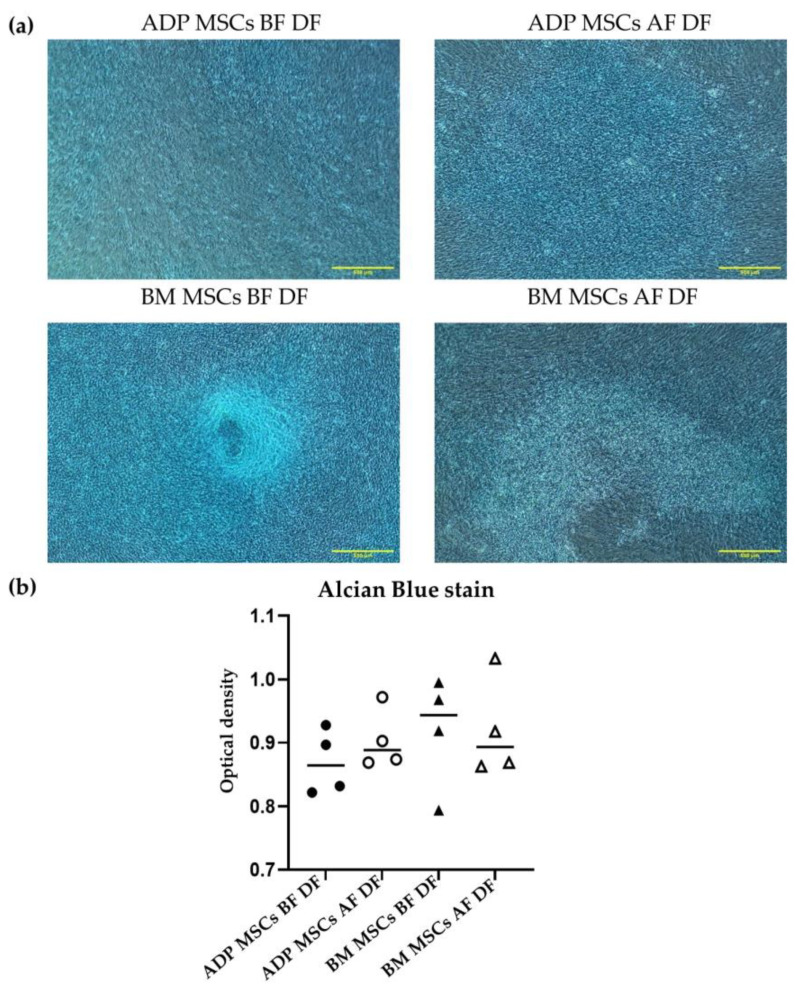
(**a**) Representative histological image of Alcian Blue staining of the induced chondrogenic differentiated cells derived from rabbits’ ADP MSCs and BM MSCs before and after cryopreservation. (**b**) The Alcian Blue stained optical density of the cartilage matrix within 6 mm^2^ of the observation area was analyzed with ImageJ. The optical densities of the stain were not significantly different. Data were analyzed with the Kruskal–Wallis test and presented as the median (*n* = 4). The scale bar represents 500 µm, magnification ×40. MSCs, mesenchymal stem/stromal cells; ADP, adipose tissue; BM, bone marrow; BF, before cryopreservation; AF, after cryopreservation; DF, differentiated cells; black circle, ADP MSCs BF DF; white circle, ADP MSCs AF DF, black triangle, BM MSCs BF DF; white triangle, BM MSCs AF DF, horizontal line, median.

The median (range) stained percentage of Alcian Blue in the ADP MSCs BF DF and BM MSCs BF DF groups was 0.9 (0.8–0.9) and 0.9 (0.8–1.0), respectively. After cryopreservation, the median (range) stained percentage of Alcian Blue in the ADP MSCs AF and MB MSCs AF groups was 0.9 (0.9–1.0) and 0.9 (0.9–1.0), respectively. There was no difference in the staining percentage with Alcian Blue staining among the groups (Figure 11).

In the gene expression analysis carried out via RT-PCR after 21 days of differentiation (Table 4 and Figure 9), *ACAN* and *CNMD* were expressed in ADP MSCs before cryopreservation (the ADP MSCs BF DF group) and in BM MSCs before and after cryopreservation (the BM MSCs BF and BM MSCs AF DF groups). *COL2A1* was expressed after differentiation in BM MSCs before cryopreservation (the BM MSCs BF DF group). *SOX9* was expressed in undifferentiated MSCs. 

## 4. Discussion

In the present study, the cell proliferation rate of ADP-derived MSCs was not altered by the cryopreservation of rabbit MSCs. Rabbit ADP-derived MSCs expressed CD9 and CD44, but the detection of CD9, a surface marker of MSCs, was decreased after cryopreservation. However, the ability to differentiate them into the three lineages was maintained, indicating that the MSCs’ function was preserved. On the other hand, the cryopreservation of bone marrow-derived MSCs decreased the proliferation rate and caused a loss of osteogenic differentiation potential. These results indicate that cryopreserved ADP-derived MSCs are more useful than cryopreserved bone marrow-derived MSCs as a source of rabbit MSCs for bone regenerative medicine research.

Bambanker is a commercially available, ready-to-use serum-free cell freezing medium. It is composed of 10% DMSO, ≤80% bovine serum albumin, and ≤10% of other medium components. This serum-free feature of Bambanker differs from the conventionally stored cells in fetal bovine serum (FBS) with DMSO. FBS has a high protein content that protects the cell from damage during cryopreservation [45]. However, unknown bovine-derived components may cause xenogenic reactions after transplantation [46,47]. Instead of FBS, the Bambanker mixture is composed of bovine serum albumin, which is the main contributor to the cryoprotective effect. Unlike conventional methods, rapid cryopreservation at −80 °C is possible without a program freezer or pre-freezing. 

One of the three minimum criteria that define MSCs is plastic adherence in standard culture conditions [48]. In this study, the morphology of plastic adherence in both rabbit ADP-derived MSCs and bone marrow-derived MSCs was a fibroblast-like spindle shape typical of MSCs. This morphology was identical to that reported for rabbit MSCs [49]. For the morphology of rabbit MSCs cryopreserved in Bambanker, no changes were observed through light microscopy at ×40 magnification.

Maintaining the cell viability and proliferative ability is one of the conditions for successful cryopreservation. In the present study, the cryopreservation of rabbit MSCs in Bambanker did not significantly reduce the cell viability of either ADP- or bone marrow-derived MSCs. In addition, there was no difference in the doubling time between ADP- and bone marrow-derived rabbit MSCs before cryopreservation. Previously reported doubling times for ADP- and bone marrow-derived rabbit MSCs were 37.45 ± 4.32 h (1.6 ± 0.2 days) [50] and approximately 5 days [5], respectively. In a long-term culture, ADP-derived human MSCs are more genetically stable and have a lower senescence rate than bone marrow-derived MSCs [51]. Lechanteur et al. [52] reported that the recovery and proliferation of cryopreserved human bone marrow-derived MSCs were very low until day 4, and then increased slightly thereafter. This aligns with the present study where the doubling time of cryopreserved ADP-derived MSCs was unchanged compared to that before cryopreservation. However, the doubling time of cryopreserved bone marrow-derived MSCs (the BM MSCs AF group) was increased compared to that of MSCs before cryopreservation (the BM MSCs BF group). These results indicate that the proliferation rate of bone marrow-derived rabbit MSCs decreased after cryopreservation.

The second minimum criterion for defining MSCs is the phenotype of MSCs [48]. Several additional immunophenotypic markers of MSCs were obtained from various sources [53,54], and it is known the immunophenotype of MSCs can change throughout culturing. In this study, flow cytometry and RT-PCR were used to examine the immunophenotypes of ADP-derived MSCs and bone marrow-derived MSCs in the 4th passage before and after cryopreservation. The flow cytometry results showed that ADP-derived and bone marrow-derived rabbit MSCs expressed CD44, a well-known surface marker for MSCs, and showed minor-level expression of the hematopoietic cell markers CD34 and CD45, confirming that these were MSCs [40,41,42,43]. Their expression percentage did not change significantly after cryopreservation. CD9 is expressed in human MSCs and MSC-derived exosomes and plays an important role in intercellular communication [44]. To the best of our knowledge, this is the first study to confirm the expression of CD9 in rabbit MSCs. The CD9 expression percentage in rabbit MSCs was high; however, its expression in ADP-derived MSCs decreased after cryopreservation compared with that before cryopreservation. This result resembles that of bone marrow-derived human MSCs. CD9 expression in bone marrow-derived human MSCs was 34% before cryopreservation and decreased to 18% after cryopreservation [55].

The RT-PCR results showed that the expression of the *CD9, CD29, CD44, CD90*, and *CD105* surface marker genes of MSCs tends to decrease after cryopreservation. Notably, *CD90* was not totally expressed after the cryopreservation of ADP-derived MSCs. *CD90* is a glycoprotein present in MSC membranes [56]. The reduction in *CD90* expression promotes osteogenesis and adipogenesis and does not affect the morphology and proliferation rate of MSCs [57]. The loss of *CD90* gene expression could have an adverse effect at the mRNA level due to cryopreservation in Bambanker or due to the progression of osteogenesis and adipogenesis of ADP-derived MSCs after cryopreservation.

The third essential criterion for defining MSCs is their ability to differentiate trilinearly into osteocytes, adipocytes, and chondrocytes [48]. We compared the multipotency of MSCs in derived cells before and after cryopreservation.

In osteogenic differentiation, ADP-derived MSCs were differentiated into osteocytes before and after cryopreservation. However, bone marrow-derived MSCs did not differentiate into osteocytes after cryopreservation. Osteogenic differentiation of ADP-derived MSCs after cryopreservation could also be associated with decreased *CD90* expression. Cryopreserved bone marrow-derived MSCs are more susceptible to intracellular damage than MSCs derived from other tissues [58,59]. Izadpanah et al. reported that the osteogenic differentiation of human bone marrow-derived MSCs was lost after passage 10 [51]. In our study, the total passage of the ADP- and bone marrow-derived MSCs used for osteogenic differentiation occurred in passage 8. This finding indicates that the loss of the osteogenic differentiation capacity may result from the source-dependent resistance to the long-time culture, together with the adverse effect of cryopreservation. 

In the osteogenesis-related gene expression, *IBSP*, a late osteogenesis marker [60], was not expressed before and after cryopreservation in both ADP- and bone marrow-derived MSCs. *RUNX2* is a transcription factor essential for osteoblast differentiation and is expressed relatively early in osteoblast differentiation [61]. *PDPN* is expressed in the late stage of osteoblasts and pre-osteocytes [62]. *OPN* is a component of the bone matrix, and its expression increases with bone mineralization [61,63]. *BMP2* induces the osteogenic differentiation of MSCs [63]. In this study, *RUNX2*, *PDPN*, *OPN*, and *BMP2* were expressed in MSCs even before the induction of differentiation, indicating that these genes cannot be used as markers of osteogenic differentiation in rabbits.

In adipogenic differentiation, bone marrow-derived MSCs after cryopreservation had a higher ability to differentiate into adipocytes than ADP-derived MSCs before cryopreservation. In the adipogenic-related gene expression, *ADIPOQ* is one of the representative adipokines produced by ADPs [64] and was expressed after the induction of adipogenic differentiation of ADP-derived MSCs before and after cryopreservation. Thus, *ADIPOQ* can be used as a marker of adipogenic differentiation of rabbit ADP-derived MSCs. *ADIPOQ* was also not expressed after the induction of adipogenic differentiation of bone marrow-derived MSCs. *PPARG* is a nuclear hormone receptor family of transcription factors, and *PPARG* and *LPL* enhance the adipogenic differentiation of MSCs [65]. In this study, however, *PPARG* and *LPL* were also expressed in MSCs before the induction of differentiation, indicating that these genes cannot be used as adipogenic differentiation markers in rabbits.

In chondrogenic differentiation, ADP- and bone marrow-derived MSCs were differentiated into chondrocytes before and after cryopreservation. However, the staining rates with Alcian Blue were very low in all groups. In the chondrogenic-related gene expression, *COL2A1* is the cartilage matrix’s major component, in addition to other proteins and proteoglycans [66]. *ACAN* is associated with cartilage matrix synthesis and, thus, is a significant structural component of articular cartilage [67]. *CNMD* is a cartilage-specific protein that stimulates the synthesis of the extracellular matrix and the growth of chondrocytes and cartilage [68]. According to the results of this study, *ACAN* and *CNMD* can be used as a marker of chondrogenic differentiation in rabbits in both ADP-derived MSCs and bone marrow-derived MSCs, and *COL2A1* can be used as a marker of chondrogenic differentiation of rabbit bone marrow-derived MSCs. *SOX9* is the first transcription factor that is essential for chondrocyte differentiation [69]. In this study, *SOX9* was expressed in both undifferentiated and differentiated MSCs. This finding was in line with the finding of Jo et al. [70], which stated that *SOX9* is expressed in stem cell pools. Since it was expressed in the undifferentiated MSCs, *SOX9* cannot be used as the chondrogenic differentiation marker.

There are some limitations to this study. First, bone marrow-derived MSCs did not demonstrate osteogenic differentiation after cryopreservation. In the future, the cryopreservation method and the passaging time of the cells to be used should be considered. Second, this study analyzed lineage-specific gene expression and showed that genes reported in humans cannot be used as differentiation markers in rabbits. We must investigate whether this is due to species differences, molecular damage from cryopreservation, or regulatory factors in gene expression. In the future, improved methods of MSC cryopreservation could be applicable not only in regenerative medicine, but also to improve fertility [71].

## 5. Conclusions

Cryopreservation in Bambanker significantly reduced the cell proliferation rate and osteogenic differentiation of bone marrow-derived MSCs, whereas those of ADP-derived MSCs remained. Moreover, cryopreservation did not significantly affect the live cell percentage in ADP- or bone marrow-derived MSCs, the surface marker expression (except CD9 expression) of ADP-derived MSCs, or adipogenic and chondrogenic differentiation. This study demonstrated that the viability, proliferation rate, and differentiation properties of ADP MSCs remained higher than that of BM MSCs after cryopreservation. We suggest that the appropriate cell sources should be chosen for a therapeutic target based on their characteristics and functional steadiness after cryopreservation.

## Figures and Tables

**Table 1:** Primer sequences.

Gene Name		Direction	Primer Sequences (5′–3′)
Surface marker genes	*CD9*	Forward	GGCTCCGATTCGACTCTCAG
		Reverse	AACCCACCAGCATCATGAGG
	*CD29*	Forward	AGAACCCTTGCACGAGTGAG
		Reverse	TCCTCCCCTCTGTCTGTGAG
	*CD44*	Forward	TTGGCGATTTCCTGGGTCTC
		Reverse	TGGTTGTGTTGTGGCTGTCT
	*CD90*	Forward	CTTCTCTGAGGCTGCTGACC
		Reverse	GCAGCACTGGGGTTCCTTAT
	*CD105*	Forward	CCACCGGCGAATACTCTCTC
		Reverse	AACAGGCCTTGGATGGTGTC
Osteogenic genes	*OPN*	Forward	AGACCCTCCCGAGTAAGTCC
		Reverse	CGGCATCGTCGGATTCATTG
	*IBSP*	Forward	TTCTGACATCACCTTGGCCG
		Reverse	TCTGCTCTCCCACTCACTCA
	*BMP2*	Forward	GGAAACGCCTCAAATCCAGC
		Reverse	TAAAAGGCGTGATACCCCGG
	*RUNX2*	Forward	CTTCAAGGTGGTAGCCCTCG
		Reverse	CCGGCCCACAAATCTCAGAT
	*PDPN*	Forward	ATGAGCCGCAGAAAACTCCA
		Reverse	CTTAGAGGAGGGAGCCGAGT
Adipogenic genes	*LPL*	Forward	CGACTGGGAACGTGTGTGTA
		Reverse	CCACACACAACCCTCTCTCC
	*PPARG*	Forward	CAAGTACGGCGTCCATGAGA
		Reverse	CGTCATGAAGCCTTGTCCCT
	*ADIPOQ*	Forward	GGTGCCTATGTCTACCGCTC
		Reverse	CGTGGTGCTGTCATAGTGGT
Chondrogenic genes	*COL2A1*	Forward	GGATAGACCCCAACCAAGGC
		Reverse	TCCACCAGTTCTTCTTGGGC
	*ACAN*	Forward	GGAACATCACTGAGGGCGAA
		Reverse	CTTCAGTCCCGTTCTCCACC
	*CNMD*	Forward	AGGAGGCTCTAGTCTGGGTG
		Reverse	TCGCCGCAGAGTTCTAAGAC
	*SOX9*	Forward	GCCCAGAAGAGCCTCAAAGT
		Reverse	TAAGAGAGGTGGGGAGGGTG
Housekeeping gene	*GAPDH*	Forward	AGCTGGTCATCAACGGGAAG
		Reverse	GAAGACGCCAGTGGATTCCA

**Table 2:** Flow cytometry analysis of the cell surface markers’ expression before and after cryopreservation in passage 4 of MSCs. Data are presented as the positive expression percentage of MSC markers (CD9 and CD44) and hematopoietic cells markers (CD34 and CD45) (median (range) (*n* = 4)). The percentage of CD9 in the ADP MSCs AF group was significantly lower than that of the ADP MSCs BF group (* *p* < 0.05). MSCs, mesenchymal stem/stromal cells; ADP, adipose tissue; BM, bone marrow; BF, before cryopreservation; AF, after cryopreservation.

Antibodies	ADP MSCs BF	ADP MSCs AF	BM MSCs BF	BM MSCs AF
CD9	92 (90–95)	41 (27–50) *	83 (22–95)	77 (41–82)
CD44	50 (32–66)	17 (10–37)	51 (29–93)	31 (22–41)
CD34	2.5 (0.8–10)	2.1 (0.7–4.0)	1.8 (0.3–6.0)	7 (4–11)
CD45	2.4 (0.6–3.0)	0.8 (0–0.9)	0.5 (0.02–1)	0.2 (0.05–0.8)

**Table 3:** RT-PCR analysis of surface marker genes’ expression in the 4th passage of MSCs before and after cryopreservation. Data are presented as the number of rabbits that expressed MSC surface marker genes (*n* = 4). MSCs, mesenchymal stem/stromal cells; ADP, adipose tissue; BM, bone marrow; BF, before cryopreservation; AF, after cryopreservation.

Surface Marker Genes	ADP MSCs BF	ADP MSCs AF	BM MSCs BF	BM MSCs AF
*CD9*	3	2	3	3
*CD29*	4	2	3	3
*CD44*	4	2	3	2
*CD90*	3	0	2	1
*CD105*	3	2	3	2

**Table 4:** RT-PCR analysis of trilineage differentiation genes’ expression in the 4th passage of MSCs before and after cryopreservation. Data are presented as the number of rabbits that expressed osteogenic, adipogenic, and chondrogenic genes (*n* = 4). MSCs, mesenchymal stem/stromal cells; ADP, adipose tissue; BM, bone marrow; BF, before cryopreservation; AF, after cryopreservation; DF, differentiated cells.

Gene		ADP MSCs				BM MSCs			
		BF	BF DF	AF	AF DF	BF	BF DF	AF	AF DF
Osteogenic gene	*OPN*	2	0	1	2	1	2	3	1
	*IBSP*	0	0	0	0	0	0	0	0
	*BMP2*	2	2	0	2	2	0	1	1
	*RUNX2*	4	4	2	4	3	3	2	3
	*PDPN*	4	4	3	4	3	4	3	4
Adipogenic gene	*LPL*	2	4	1	2	2	0	2	2
	*PPARG*	3	0	0	2	2	3	0	0
	*ADIPOQ*	0	4	0	2	0	0	0	0
Chondrogenic gene	*COL2A1*	0	0	0	0	0	4	0	0
	*ACAN*	0	3	0	0	0	4	0	1
	*CNMD*	0	2	0	0	0	2	0	1
	*SOX9*	3	2	2	4	2	4	1	4

## Data Availability

Data are contained within the article.
